# Topical treatment of oral lichen planus with anthocyanins

**DOI:** 10.4317/medoral.19472

**Published:** 2014-06-01

**Authors:** Emilce Rivarola de Gutierrez, Amanda Di Fabio, Susana Salomón, Héctor Lanfranchi

**Affiliations:** 1 Facultad de Odontología, Universidad Nacional de Cuyo, Mendoza, Argentina; 2 Facultad de Ciencias Médicas, Universidad Nacional de Cuyo, Mendoza, Argentina; 3 Universidad Juan Agustín Maza, Mendoza, Argentina; 4 Facultad de Odontología, Universidad de Buenos Aires, Argentina

## Abstract

Background: Oxidative stress is involved in oral lichen planus (OLP) pathogenesis; meanwhile anthocyanins are natural antioxidants present in grapes skin. Objectives: The aim of this research was to verify the utility of anthocyanins, extracted from grapes skin, for the local treatment of oral lichen planus and to compare it with clobetasol propionate- neomycin -nystatin cream (CP-NN). Study Design: Prospective, non-randomized study, with control group. Fifty-two patients with OLP were included. We divided patients into two categories: erosive oral lichen planus (EOLP) and non erosive oral lichen planus (NEOLP). 38 had EOLP (17 cases and 21 controls) and 14 presented NEOLP types (9 cases and 5 controls).Cases received local treatment with anthocyanins from grapes and controls, were treated with CP-NN. The clinical evolution of patients was followed up during six months. Results: The patients had a therapeutic response with anthocyanins. This was better than CP-NN treatment for patients with EOLP, in improving the involvement score of the oral mucosa and in the morphometric study of the affected areas. In EOLP there were no statistically significant differences in: therapeutic response time, the evolution of pain, or the relapse rate between the two groups. With respect to the treatment of NEOLP there was improved pain relief in the group treated with anthocyanins. This was not observed with CP-NN. The resting analized variables showed no significant difference with both treatments. Conclusions: OLP has a favorable response to local treatment with anthocyanins from grapes. We found an equal to or better response than with CP-NN treatment. Many of our patients have systemic diseases, which may contraindicate the use of steroids. With regard to this particular group, the use of this natural antioxidant present in the diet is considered advantageous.

** Key words:**Anthocyanins, antioxidants, chemoprevention, morphometry, oral lichen, oxidative stress.

## Introduction

OLP is a chronic disease of unknown etiology and autoimmune pathogenesis. The presence of oxidative stress in OLP has been demostrated. It is caused by an imbalance between the production of reactive oxygen species (ROS); and the ability of tissues to neutralize the reactive intermediates or repair the resulting damage (1). The formation of lesions with potentially mutagenic DNA due to oxidative stress, related to inflammation, may contribute to the development of oral cancer in OLP (2,3). It occurs in atrophic, bullous and erosive OLP, but not usually in reticular clinical forms. Based on these pathophysiological observations, we decided to perform a therapeutic experience with topical antioxidants in patients with OLP. The extracts of grape seeds and grape skins contain anthocyanins (from greek anthos: flower and kyanos: blue) (4). Anthocyanins are considered “the main antioxidants of the plant kingdom” as they block the spread of free radicals (5). These are polyphenolic groups which are present also in other fruits, vegetables, chocolate, tea, among others. An average person consumes 180-1000 mg. / day of these substances (6).

## Material and Methods

The present study was approved by the Ethic Committee of the Lagomaggiore Hospital of Mendoza, Argentina. Written informed consent was obtained from each subject. Fifty two patients with all clinical types of OLP were included in this study. To achieve a more accurate comparison, patients were subdivided into EOLP and NEOLP forms. In the group EOLP erosive and bullous forms were included. NEOLP comprised reticular, keratotic, pigmentary and atrophic OLP forms. Biopsy was performed on 45 patients. In two of them results were nonspecific and the rest confirmed diagnosis.

Inclusion and exclusion criteria: patients with clinical manifestations of OLP were included in this study. Patients younger than 16 years old and patients with antecedents of organ transplants were excluded.

Study design: Prospective, non-randomized study, with control group. All patients of this study received treatment for dental septic foci and were educated in teeth brushing techniques. We divided patients into two categories: EOLP and NEOLP clinical forms. These categories were then divided into two groups each one: cases, with local treatment with anthocyanins from grapes, and controls, treated with CP-NN. The clinical evolution of patients was followed up over six months. For the cases group we indicated natural red colorant 0352 Christian Hansen Laboratories Argentina. This is used as a colorant in the food industry. It is a violet-colored powder. Its ingredient is purified anthocyanin from grapes, Vitis vinifera. Anthocyanins were administered in 100 mg./doses diluted in 5 ml of water, mouth rinses, during 5 minutes and spit, three times a day. This amount, is lower than the average dose usually ingested (6,7). Furthermore this quantity was compared in vitro with the equivalent of 225 mg of vitamin E, and showed higher scavenger effect on ROS (8,9). The control group received: CP-NN cream (100 g of commercial preparation containing: 17-clobetasol propionate (micronized) 0.050 g, Neomycin (as sulfate) 0.350 g; Nystatin (micronized) 100.000 U / g. This was applied three times daily locally on lesions. The follow up was protocolized and there were considered among other parameters: epidemiological data, presence of systemic diseases and treatments, degree of pain, graded from 0 to 10 and measured through verbal analogue scale, days to achieve improvement, relapse, measurement of the involved areas and dental indices. We performed a total compromise score dividing mouth into 14 sectors and evaluating each one as: Normal= 1, moderate injury= 2 and serious injury= 3 ( Table 1). A morphometric study of the lesions was performed. Image Pro Plus (software and image analyzer processor) was used to examine the images obtained during examinations. Affected areas were compared at the initial and improvement consultations in cases and controls.

**Table 1:**
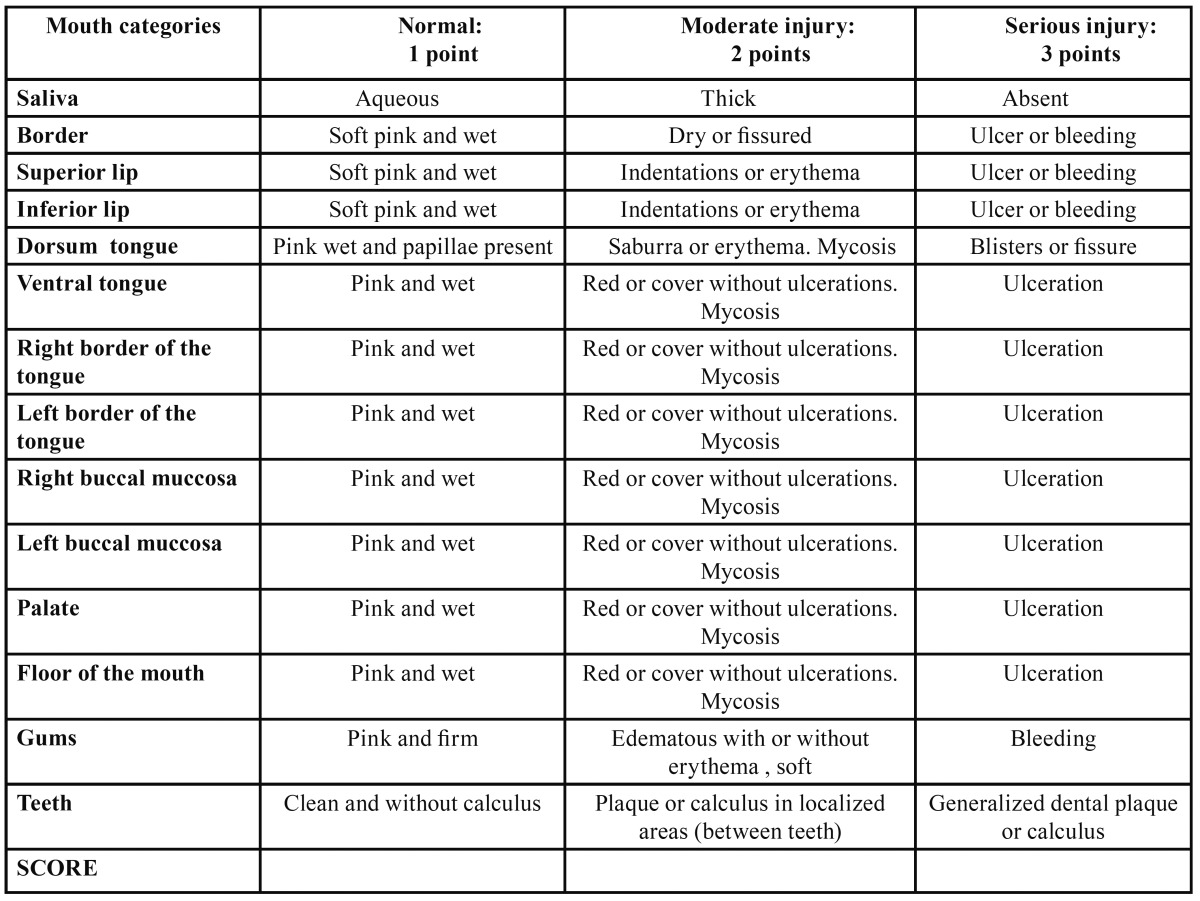
Total compromise score. Mouth is divided into 14 sectors or categories and each one is evaluated as: Normal= 1, moderate injury= 2 and serious injury= 3.

Epidemiological data were analyzed using Epi-info. This included an analysis of central tendency and dispersion measures, Student’s t test and Fisher exact test. The morphometric analysis was analyzed with GraphPad InStat 3.0 and GraphPad Prism version 5. We used Fisher’s exact test, Wilcoxon test, Mann-Whitney, ANOVA, Kruskal-Wallis and Friedman test as appropriate. A value of *p* ≤ 0.05 was considered significant.

## Results

Fifty-two patients with OLP were included. 38 had EOLP (17 cases and 21 controls) and 14 presented NEOLP types (9 cases and 5 controls). The average age was 61.6 years (SD ± 10.6), 87.5% were women. In 77.1% of them (95% CI 62.69 to 87.97) the evolution of OLP was over one year. 87.5% (95% CI 74.75 to 95.27) had also other diseases. 71% of patients ingested three or more drugs, The main drugs included antihypertensives (inhibitors of angiotensin converting enzyme, beta blockers and calcium channel blockers), thyroid hormones and antidepressants among others. 53.85% had amalgam restorations, and in two patients a history of B hepatitis was found. Caries accounted for 50% of cases and 48% of controls. 60% and 78% of patients used dentures in respective group. Plaque was present in 76% of cases and 83% of controls and calculus were 68% and 52% respectively.

There was no significant difference between patients who had amalgams and those without them (*p*= 0.36), neither between EOLP and NEOLP forms (*p*= 0.32) at baseline. The difference between means, in the number of days to reach improvement, was not statistically significant (*p*= 0.63), neither in patients undergoing removal of amalgams or those who remained with the same restorations (*p*= 0.18).

**Figure 1:**
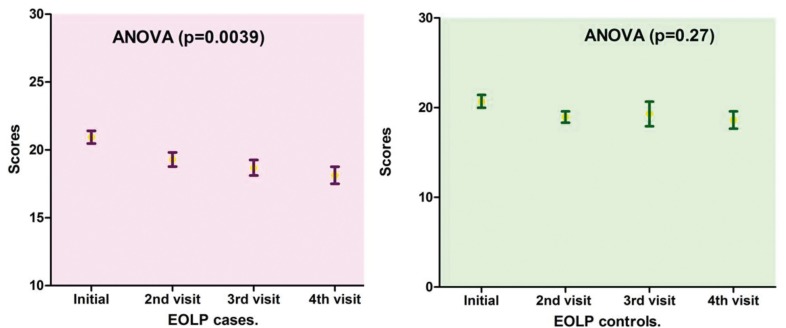
Evolution of scores in patients with EOLP with both treatments. In patients treated with anthocyanins there was significant difference when lesions were evaluated at the initial consultation and three subsequent visits. In the control group this difference was not demonstrated in the follow up scores.

Oral compromise scores in patients with EOLP exhi-bited no significant difference initially between groups (*p*= 0.79). The difference of initial scores, paired with the visit of improvement was significant (*p*= 0.01) in cases group.There was also a significant difference seen with ANOVA when lesions were evaluated at the initial consultation and three subsequent controls (Fig. 1) (*p*= 0.0039). In the control group with EOLP the scores compared between initiation and improvement, showed significant difference (paired t test, *p* = 0.0008) also. This difference was not demonstrated in the follow up scores (Fig. 1) (ANOVA, *p*= 0.27). In patients with NEOLP scores of initial control matched with the improvement visit (paired t test), showed no significant difference for cases (*p*= 0.99) neither for controls (*p*= 0.62). In these patients with NEOLP no significant differences were seen with ANOVA comparing the initial and subsequent consultations; in both groups: cases (*p*= 0.71) and controls (*p*= 0.06).

In patients with EOLP and NEOLP forms we found no significant difference in the degree of pain at baseline between the groups. Matched analysis of pain at the first visit and at the time of improvement showed significant difference in cases (*p*= 0.0001) and in controls (*p*= 0.0007). The comparison showed no significant differences neither in the improvement of the degree of pain nor in the time to improve with both treatments. In patients with NEOLP lichen, the cases group demonstrated significant difference (*p*= 0,03) comparing the initial and the improvement control. In the control group, there was no significant difference (*p*= 0.24) between baseline and the recovery consultation.

The final consultation was performed on 27 patients with EOLP lichen. The group included 14 cases and 13 controls.It was seen that the response was good in 12 of 14 (85%) in the cases group and in 9 of 13 (69%) of the control group (*p*= 0.64) The response was poor in 14% of the case group and 23% in the control group (*p*=0.38). The mean treatment time was 164 days (DS±70.8) in the case group and 145.7 (DS±77.9) in the controls. No significant differences were seen between both groups (*p*= 0.53).

In patients with NEOLP there was no significant difference in the number of days since the first consultation to final inspection, neither in the lesions and symptoms improvement (*p*= 1.00) comparing both treatments.

**Figure 2:**
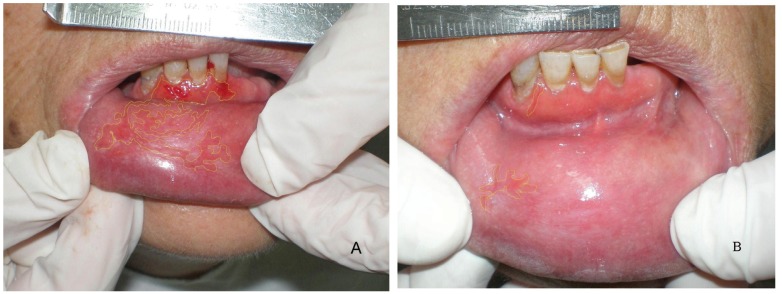
Morphometric study. Affected areas at baseline versus the affected areas in the clinical assessment of improvement, in a patient, with EOLP treated with anthocyanins. A: date: 18-12-06 and B: evolution: 16-01-07.

**Figure 3:**
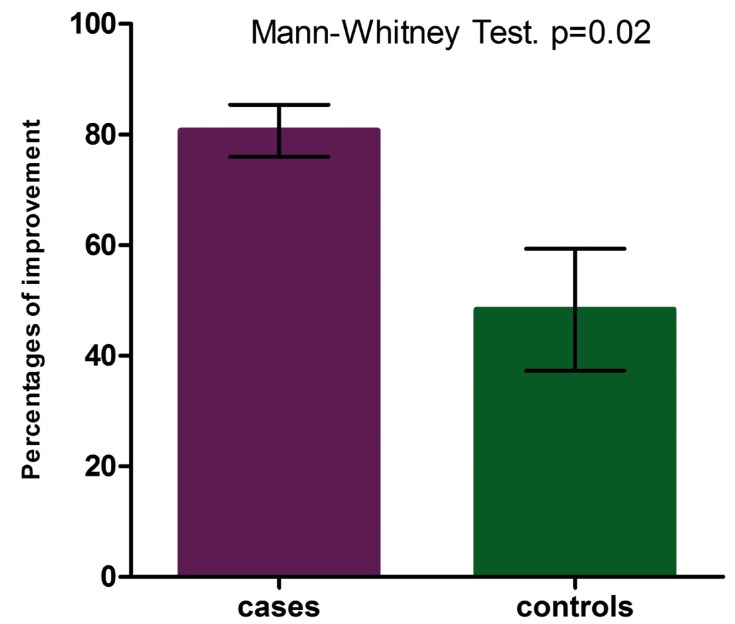
Percentages of improvement in EOLP. In patients with EOLP comparing the percentages of improvement in the measured areas, it was greater for the case group (*p*=0.02).

Morphometric study: The comparison of the affected areas at baseline did not show significant difference neither in EOLP (*p*= 0.49) nor in NEOLP (*p*= 0.31). In the EOLP cases group, analyzing the affected areas at baseline versus the affected areas in the clinical assessment of improvement (Fig. 2,  Table 2), with the Wilcoxon test for paired samples, we encountered *p* <0.0003, considered extremely significant. In EOLP control group ( Table 2): we found *p* = 0.0035, considered very significant. In EOLP comparing the percentages of improvement in the measured areas, it was greater for the case group (*p*=0.02) (Fig. 3). In patients with NEOLP ( Table 3) with Student’s t test for paired samples, we did not find significant difference in the areas affected at the beginning and end of treatment in NEOLP cases(*p*= 0.13) and in controls (*p*= 0.30).

**Table 2:**
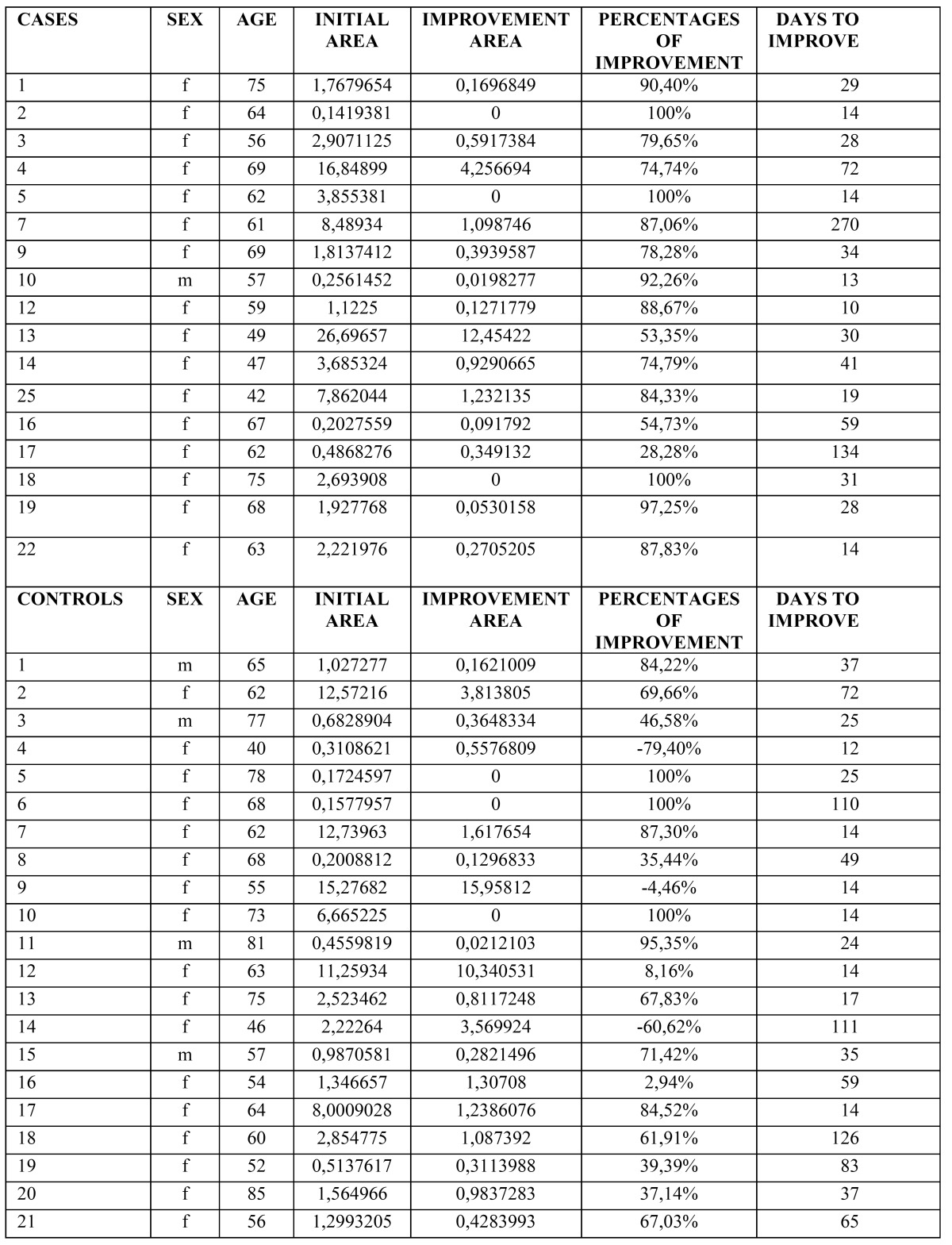
EOLP. Measured areas and days to improvement.

**Table 3:**
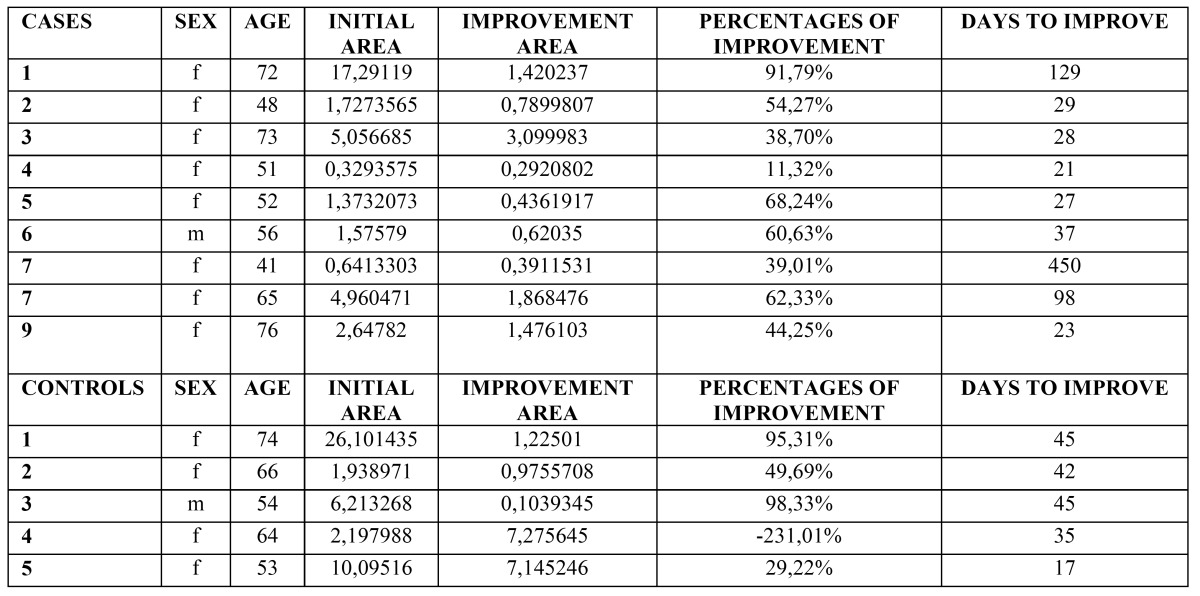
NEOLP. Measured areas and days to improvement.

Time to reach the improvement: ( Table 2) for patients in EOLP case group the average to achieve improvement was 49 days (SD ± 64.39). In the control group mean to achieve the improvement was 45 days (36.10 ± DS).There was no significant difference between the two groups (*p*= 0.82). Regarding NEOLP ( Table 3) no significant differences were found between groups for reaching improvement either (*p*=0.38).

## Discussion

This work was designed and aimed at treating OLP with anthocyanins, a natural antioxidant substance that simultaneously has proven to have no toxic effects.

In our patients, the most frequent concomitant diseases were hypertension, depression, diabetes and hypothyroidism. To date, local corticosteroids are of choice, but absorption through the mucosa has been demonstrated causing inhibition of the hypothalamic pituitary adrenal axis (10). Corticosteroid systemic effects are known to increase blood pressure, induce psychosis and elevate blood glucose levels among others. Another medication, with the same local therapeutic effects of corticosteroids would be very usefull specially if it does not damage the health of patients. In relation to the known treatments and oral cancer, tacrolimus has been related to carcinogenic potential and this leads us to reconsider the benefits of its application, particularly because OLP has malignant potential especially in erosive and atrophic forms (11).

Anthocyanins modulate apoptosis and signals that provide links between inflammation and cancer, and further regulate tumor angiogenesis and invasiveness (12). Numerous reports demonstrate anthocyanins high bioavailability and their protection against diseases induced by free radicals such aging and carcinogenesis (13-15). In our clinical research, we demonstrated the usefulness of anthocyanins therapy for the treatment of OLP, especially in erosive forms, which are known to be potentially malignizables.

Caries, dentures, dental plaque and calculus, are dental condition present in our patients and they are not favorable for the use of local corticosteroids due to its immunosuppressive effect. Grapes polyphenols inhibit growth of *Escherichia coli*. *Staphylococcus aureus, and Candida albicans* (16). So the anti-inflammatory effect of anthocyanins could add antibacterial and antifungal properties (17,18). Similarly, those effects were added to the control group through neomycin and nystatin.

When evaluating compromise scores along the follow up, we found that patients with EOLP, cases and controls, improved their scores significantly in both groups. There was no statistical difference in the number of days until the scores improved. On subsequent visits, it was recorded that in those treated with anthocyanins improvement continued increasing and there were significant differences when analyzing the first and the three following queries. Meanwhile we could not demonstrate significant differences in the control group between the first consultation and the three later. We consider this find of great importance because it shows statistically significant, better therapeutic results with anthocyanins. It is considered to occur in EOLP, which are already known to be potentially malignizables, and corticosteroids can have even an immunosuppressive effect in these patients. In patients with NEOLP no significant differences were found along the follow up with the both treatments. It is relevant to have found that treatment with anthocyanins has been found to have the same effect as realized with the CP-NN, which is the highest potency corticosteroid known and is the gold standard local treatment.

Conclusions: OLP has a favorable response to local treatment with anthocyanins from grapes. In patients with EOLP an equal to better response than with CP-NN treatment was noted.

The presence of amalgams did not influence the clinical course of our patients. The compromise scores showed that EOLP patients progressively improved with anthocyanins along the course of follow up. In NEOLP patients there was no significant difference. Regarding the pain, although there is a tendency to a better response with anthocyanins, in EOLP there was no significant difference between groups. In NEOLP patients the response of pain was significantly better with anthocyanins. We found no significant difference between groups in the time to reach improvement and the percentage of relapses. In the morphometric analysis of patients with EOLP the percentage of improvement was higher for the group cases. In patients with NEOLP no differences between treatments were found There were no side effects related to local treatments performed during this work. Many of our patients have other diseases, which may contraindicate the use of steroids. Especially in these persons, it is considered to be an advantage in the use of this natural antioxidant present in grapes. There is extensive literature linking anthocyanins with chemoprophylaxis of diseases related to oxidative stress. New contributions are necessary to consider the effect of anthocyanins in other oral inflammatory conditions, especially in those with chronic course or malignant potential, where oxidative stress plays a role in the pathogenesis.
